# Soil organic carbon accumulation during post-agricultural succession in a karst area, southwest China

**DOI:** 10.1038/srep37118

**Published:** 2016-11-23

**Authors:** Liqiong Yang, Pan Luo, Li Wen, Dejun Li

**Affiliations:** 1 Key Laboratory of Agro-ecological Processes in Subtropical Region, Institute of Subtropical Agriculture, Chinese Academy of Sciences, Changsha 410125, Hunan, China; 2 Huanjiang Observation and Research Station for Karst Ecosystems, Institute of Subtropical Agriculture, Chinese Academy of Sciences, Huangjiang 547100, Guangxi, China; 3 University of Chinese Academy of Sciences, Beijing 100049, China

## Abstract

This study was aimed to investigate the direction and magnitude of soil organic carbon (SOC) dynamics and the underlying mechanisms following agricultural abandonment in a subtropical karst area, southwest China. Two post-agriculture succession sequences including grassland (~10 years), shrubland (~29 years), secondary forest (~59 years) and primary forest with cropland as reference were selected. SOC and other soil physicochemical variables in the soil depth of 0–15 cm (representing the average soil depth of the slope in the studied area) were measured. SOC content in the grassland was not significantly elevated relative to the cropland (42.0 ± 7.3 Mg C ha^−1^). SOC content in the shrubland reached the level of the primary forest. On average, SOC content for the forest was 92.6 ± 4.2 Mg C ha^−1^, representing an increase of 120.4 ± 10.0% or 50.6 ± 4.2 Mg ha^−1^ relative to the cropland. Following agricultural abandonment, SOC recovered to the primary forest level in about 40 years with a rate of 1.38 Mg C ha^−1^ yr^−1^. Exchangeable Ca and Mg were found to be the strongest predictors of SOC dynamics. Our results suggest that SOC content may recover rapidly following agricultural abandonment in the karst region of southwest China.

The soil organic carbon (SOC) pool is much greater than the sum of C stored in plants and the atmosphere^1^. Hence even trivial dynamics in SOC pool may have a great impact on atmospheric CO_2_ level and subsequently the global climate^2^. Land use change has been recognized a major driver of SOC dynamics. Conversion of natural ecosystems to cropland may deplete SOC pool by as much as 75% (with most between 30–50%) dependent on climate zones and ecosystem types^2^,^3^. It has been estimated that the historic SOC loss is about 50 to 100 Pg, with most lost as CO_2_ into the atmosphere^3^. However, the processes of SOC loss can be reversed, i.e., soil acts as a net sink for atmospheric CO_2_, when cropland is converted to grassland or forest^4^,^5^.

In spite of many years of studies, much uncertainties still exist in terms of the rate of SOC accumulation and the underlying mechanisms following agricultural abandonment or afforestation. SOC has been found to increase, decrease, change insignificantly, or decrease initially followed by a gradual increase^6^,^7^. According to a literature survey, the rate of absolute SOC content change may vary from less than −5 Mg C ha^−1^ yr^−1^ (1 Mg = 10^6^ g) to greater than 5 Mg SOC ha^−1^ yr^−1^ 5. SOC sequestration following afforestation is apparently related to prior land use, climate, tree species planted and afforestation time^5^. Nevertheless, the controls of soil variables on SOC sequestration have not been well understood so far.

SOC sequestration is primarily determined by soil organic matter input and SOC decomposition rate^5^. The decomposition rate is related to SOC stability, which is controlled by soil physiochemical properties including soil texture, soil aggregates and mineral characteristics^8^,^9^,^10^. In general, the regulation of soil properties on SOC stability can be roughly categorized into two major mechanisms, i.e., (i) occlusion in aggregates, and (ii) stabilization by interaction of SOC with mineral surfaces or polyvalent cations^9^,^10^. The major polyvalent cations are Ca^2+^ and Mg^2+^ in neutral and alkaline soils^10^. More SOC has been found to accumulate in calcareous (with more Ca^2+^ and Mg^2+^) than non-calcareous soils, regardless of whether the calcium occurred naturally or amended artificially^8^,^11^. In the other hand, soil texture especially clay and silt content has often been related to SOC stability and content^12^,^13^,^14^,^15^,^16^,^17^,^18^. The relative importance of the above soil variables in SOC sequestration varies among eco-regions and has not been well understood. This kind of knowledge is undoubtedly crucial for understanding the mechanisms underlying SOC sequestration and for improving SOC cycle models.

Over the past decades, China has implemented several national-scale ecological restoration programs including the so-called “Grain for Green” project (GGP). These ecological restoration programs have been proposed to substantially enhance SOC accumulation^7^,^19^,^20^,^21^,^22^. For example, according to a recent meta-analysis, cumulative soil C sequestration due to GGP was 156 ± 108 Tg C (95% CI hereafter) over the period of 1999 to 2012 with a mean accumulation rate of 12 ± 8 Tg C yr^−1^, and will increase to 383 ± 108 Tg C by 2050^19^. However, these estimates may be rough since the regional distribution of measurements is unbalanced with, for example, very limited measurements conducted in the vast calcareous karst region of southwest China. This region has an area of about 0.51 million km^2^ of contiguous exposed/outcropped carbonate rock areas (or karst areas), accounting for 5.8% of the national land^23^. The region is famous for the extremely fragile geological background and susceptibility to land degradation upon human disturbances, particularly agricultural activities. During the past decades, a large portion of the karst region in southwest China have been degraded due to SOC loss and soil erosion following destruction of natural vegetation and arable cultivation. Under the supports of GGP and other ecological restoration projects, most of the degraded land have been converted into woodland or grassland so far. Nevertheless, whether the vegetation restoration in this karst region will lead to substantial C sequestration is still not clear.

In the present study, two succession sequences including grassland, shrubland, secondary forest and primary forest were selected with cropland as reference in a calcareous kart region in southwest China. The grassland, shrubland and secondary forest were developed from abandoned cropland. In a companion paper, we reported that soil nitrogen (N) was recovered in about 67 years following agricultural abandonment^24^. N release from bedrock was suggested as an important N source besides atmospheric N deposition and biological N fixation in the studied area^24^. Due to similar patterns of SOC and soil N accumulation during ecosystem development^5^,^25^, we hypothesized that SOC would also recover to the primary forest level in a similar timescale as soil N. In addition, soil Ca^2+^ and Mg^2+^ levels in the cropland were decreased due to elevated erosion and biomass harvesting which moved Ca and Mg away from the system, but would be rapidly replenished following agricultural abandonment owing to the rapid weathering of Ca and Mg rich bedrock in the karst region. As interaction with soil minerals via polyvalent cations is a major way for SOC stability and the major polyvalent cations are Ca^2+^ and Mg^2+^ in neutral and alkaline soils^10^, we hypothesized that exchangeable Ca and Mg would exert a strong control on SOC accumulation.

## Results

### SOC contents and their dynamics with succession

**Figure 1:**
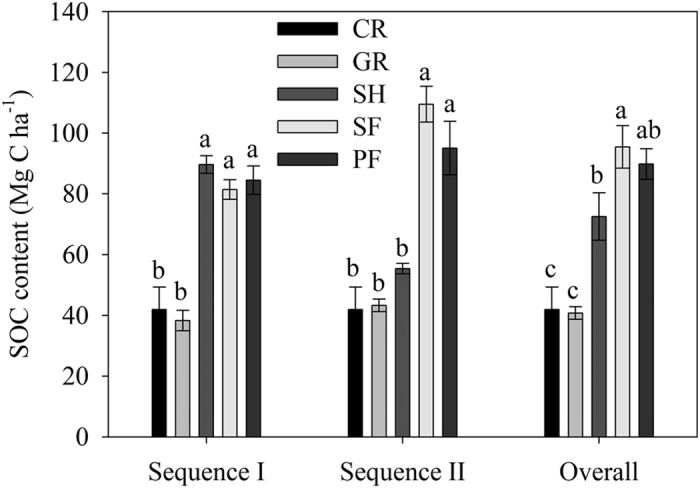
Variation of SOC content along post-agriculture succession for sequence I, sequence II and the overall data set (CR, cropland; GR, grassland; SH, shrubland; SF, secondary forest; PF, primary forest). Bars represent mean ± standard error. Different letters represent significant difference among successional stages at *p* < 0.05 level.

There was a significant effect (*p* < 0.0001) of succession stage on SOC content, but the effect of sequence on SOC content were not significant (*p* > 0.05). SOC content for the depth of 0–15 cm was 42.0 ± 7.3 (mean ± standard error) Mg C ha^−1^ at the cropland. For both sequences, SOC levels at the grassland were not elevated significantly relative to the cropland. However, SOC contents at shrubland (89.6 ± 2.9 Mg C ha^−1^) were significantly increased and equivalent to levels of the secondary forest (81.4 ± 3.3 Mg C ha^−1^) and primary forest (84.5 ± 4.7 Mg C ha^−1^) for sequence I (Fig. 1). For sequence II, SOC content at shrubland was not statistically different from the cropland or grassland, but that of secondary forest (109.5 ± 5.9 Mg C ha^−1^) approached the SOC level of the primary forest (95.1 ± 8.8 Mg C ha^−1^).

Overall, SOC level at the grassland was not elevated significantly relative to the cropland. However, SOC content in the shrubland (72.5 ± 7.8 Mg C ha^−1^) was significantly increased and was not significantly different from that in the primary forest (Fig. 1). SOC content in the secondary forest was 95.4 ± 7.0 Mg C ha^−1^, which was significantly greater than that in the shrubland, but was in the same level with the primary forest (89.8 ± 5.0 Mg C ha^−1^). SOC contents in the forests represented an increase of 120.4 ± 10.0% or 50.6 ± 4.2 Mg ha^−1^ relative to the cropland.

**Figure 2:**
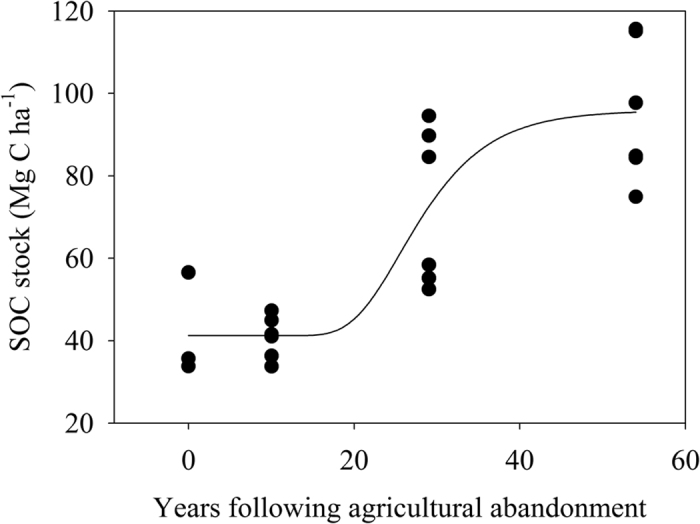
Relationship between SOC content and years following agricultural abandonment (y = 41.23 + 54.64(1 − exp(−0.17×))^76.28^, r^2^ = 0.75, *p* < 0.0001, n = 21). Each point denotes the mixture of 10 soil samples in a plot.

SOC dynamics with years following agricultural abandonment could be well fitted with a Chapman function (Fig. 2). According to the fitting, the time needed for SOC content to reach 95% of steady state level was about 40 years. The average rate of SOC accumulation over the period of recovery was 1.38 Mg C ha^−1^ yr^−1^.

### Controls on SOC dynamics

**Figure 3:**
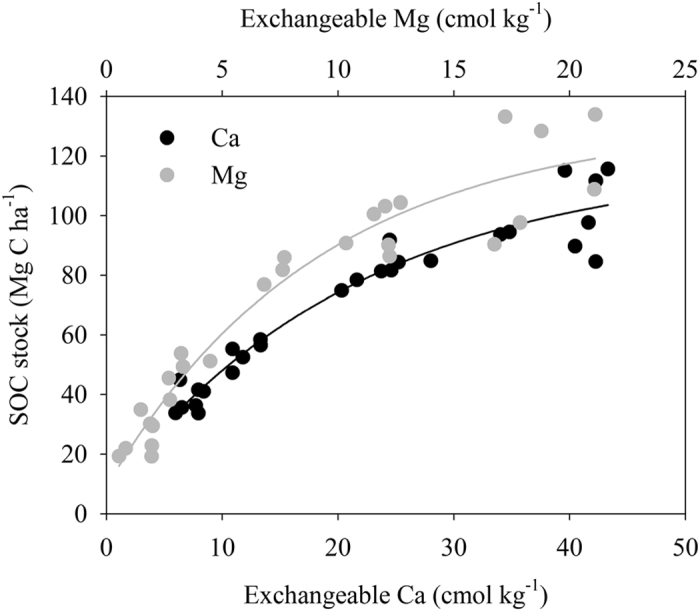
Relationship between SOC content and exchangeable Ca (y = 6.14 + 112.20(1 − exp(−0.05×), r^2^ = 0.93, *p* < 0.0001, n = 27) or Mg (y = 26.49 + 87.43(1 − exp(−0.11×), r^2^ = 0.93, p < 0.0001, n = 27). Each point denotes the mixture of 10 soil samples in a plot.

According to the Pearson’s correlation coefficients, most of the soil variables were positively related to SOC contents except exchangeable Na (which was not significantly related to SOC) and clay (which was negatively related to SOC) (Table 1). Exchangeable Ca and Mg were found to have the greatest coefficients. Further analysis revealed that SOC content was exponentially related to exchangeable Ca or Mg (Fig. 3).

**Figure 4:**
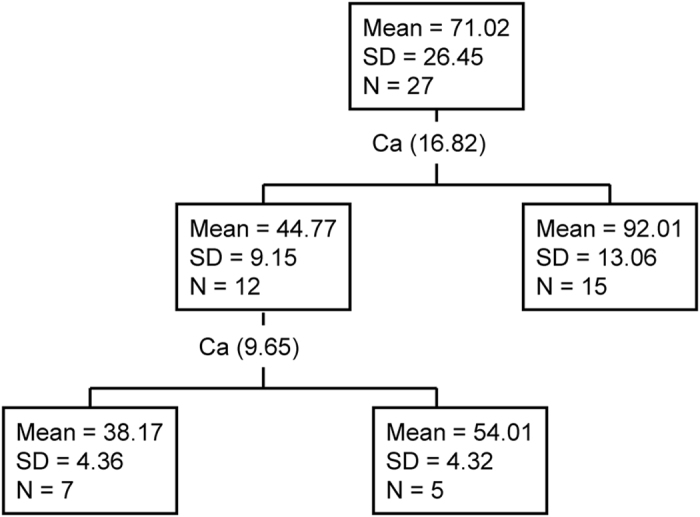
Classification and regression tree (CART) showing how predictor variables explain SOC variance (86% variance explained).

**Figure 5:**
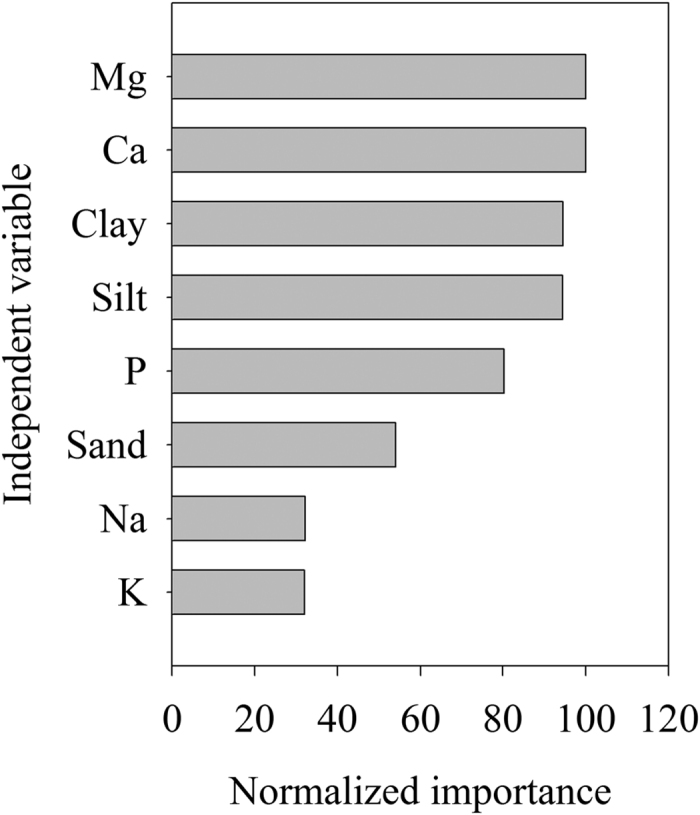
Normalized importance of each explanatory variable to SOC variance according to classification and regression tree analysis (CART).

Stepwise MLR analyses revealed that the selected variables explained 94% of SOC variance. Exchangeable Ca was the strongest explanatory variable and along explained 89% of SOC variance (Table 2). Clay and exchangeable K were the two strongest explanatory variables next to exchangeable Ca for SOC dynamics (Table 2). Classification and regression tree analysis provides another evidence supporting that Ca and Mg were the strongest predictors for SOC variance (Fig. 4). They both contributed equally to SOC dynamics (Fig. 5). Clay and silt were the strongest predictors next to Ca and Mg. All the variables explained about 86% of SOC variance.

## Discussion

### Rate of SOC content accrual

In our study, significant increase in SOC content was not found until the shrubland stage (about 29 years after agricultural abandonment across the two sequences). This is consistent with a meta-analysis, which shows that SOC content is found to be significantly increased about 30 years following afforestation^5^. SOC contents in the forests were increased by 120.4 ± 10.0% relative to the cropland in the current study. Conversion of cropland to forest resulted in 46–69% increase in SOC content in the 0–20 cm depth in China^19^,^21^, while led to 29–53% increase over the world^4^,^26^ according to meta-analyses. A literature survey revealed that most studies encountered 35–100% increase in SOC contents following reforestation or afforestation of cropland^27^. These facts indicate that SOC content increase in the current study is at the high end of the literature reported range. Shi and Han^19^ reported that the mean rate of SOC accumulation was 1.54 Mg C ha^−1^ yr^−1^ for natural succession from cropland over China, while the mean rates ranged from 0.57 to 0.97 Mg C ha^−1^ yr^−1^ for afforestation of cropland in south China and southwest China; and the rates of relative content change varied from 1.2 to 10.7% yr^−1^ for natural succession across China. It seems that the rates of SOC accumulation in the current study were within the reported range of China.

Inconsistent with our first hypothesis, SOC content recovered to level of the primary forest within a much shorter duration (40 years) relative to soil N, which was recovered in about 67 years following agricultural abandonment^24^. Recuperation of SOC contents to pre-deforestation levels in decadal scales have been observed by a few studies^28^,^29^. For example, SOC content recovered to pre-deforestation level within 20 years following afforestation of cropland in the lower montane region of northwestern Ecuador^29^. But quite a few studies reported that SOC contents decreased or increased little within the initial several decades following agricultural abandonment^5^,^30^. In fact, the time for recovering to pre-deforestation levels are generally very long. For example, Poeplau, *et al*.^31^ reported that the time for SOC of mineral soils to reach pre-deforestation levels after afforestation of cropland was estimated to be greater than 120 years. This suggests that the recovery of SOC in the studied area is relatively rapid.

### Controls on SOC dynamics

Cumulating evidence has shown that SOC in fine silt and clay fractions is more stable than that in other soil fractions^10^. The proportion of silt and clay particles has been proposed as the determinant of capacity of soils to preserve SOC^14^, and is used to assess regional SOC sequestration potential^18^,^32^. However, clay and silt were not the strongest explanatory variables of SOC dynamics in the current study. In fact, although soil texture especially clay has often been related to SOC sequestration^14^, much of the evidence for direct effect of clay on SOC comes from short-term laboratory incubations^12^. In a field survey over New Zealand, soil clay explained little of the variation in soil C dynamics across all soils and within each soil type^12^.

Consistent with our second hypothesis, our study revealed that exchangeable Ca and Mg was the strongest predictors for SOC dynamics with succession. A few studies have reported that limestone addition or increased soil Ca level promote SOC sequestration^9^,^11^,^33^,^34^. A study in Rothamsted Farm found that SOC accumulation rate in the field which was chalked a century ago was more than two times that in the adjacent field not chalked before^11^. The authors proposed that the presence of free CaCO_3_ likely slowed SOC decomposition^11^. Nevertheless, Clough and Skjemstad (2000) found that CaCO_3_ per se did not protect soil organic matter against degradation while exchangeable Ca likely decreased SOC decomposition because any free SOC would rapidly bond with the excess Ca ions. The bond of SOC with Ca is probably through polyvalent cation bridges. The major polyvalent cations occurred in soil are ions of Ca and Mg in neutral and alkaline soils^10^. Similar to our study, the positive effect of exchangeable Ca or Mg on SOC accumulation have been found in other studies^8^,^9^. In addition, the exponential relationship between SOC and Ca or Mg implied that SOC content would not increase linearly with Ca or Mg, but would reach a maximum level.

### Concluding remarks

Our study indicated that SOC could be recovered to the primary forest level in about 40 years with a rate of 1.38 Mg C ha^−1^ yr^−1^. Exchangeable Ca and Mg were found to be the strongest predictors of SOC dynamics in this calcareous karst region. These findings have great implications for ecological restoration in the karst region of southwest China, where most of the degraded land has been converted into woodland or grassland over the past decades. According to our study, the agricultural abandonment and the following succession in the karst region were likely accompanied by rapid SOC accumulation. Nevertheless, only two succession sequences were investigated here, to better assess the effects of ecological restoration on SOC dynamics over a broader karst area, more relevant studies are undoubtedly needed.

## Materials and Methods

### Site description

This study was conducted at Mulun National Nature Reserve (107°53′–108°05′E, 25°06′–25°12′N) in Guangxi Zhuang Autonomous Region, southwest China. This region is located in the subtropical humid forest life zone with a monsoon climate. Annual mean relative humidity is greater than 80%. Mean annual air temperature is 15.0–18.7 °C, with the lowest monthly mean in January (3.4–8.7 °C) and the highest in July (23.0–26.7 °C). Mean annual precipitation ranges from 1530 to 1820 mm with a distinct seasonal pattern. The period from April to August is a wet season and that from September to March is a dry season. The studied areas are characterized by a typical karst landscape with gentle valleys flanked by steep hills. The bedrock is mostly limestone nested with dolomite. The soil is calcareous lithosols (limestone soil) according to the FAO/UNESCO classification system^35^. Soil depth varies from 0 to 80 cm in the valley and ranges from 0 to 30 cm on the slopes.

Mulun National Nature Reserve has an area of 10829.7 ha and was established in 1991 in order to protect the remnant primary forests for the calcareous karst region, southwest China. The primary forests, usually evergreen and deciduous mixed forests, are located in the core zone of the reserve. In the outer zone of the reserve, many vegetation types in various successional stages are distributed.

### Field sampling

Two succession sequences were identified. The criteria for selecting the two sequences include 1) the age for a given stage between the two sequences are the same; 2) the geochemical background and thus soil types are similar; 3) the species composition for a given stage between the two sequences are different in order to detect whether succession with different species plays a role in determine SOC sequestration; 4) the aspect and slope are different for the two successions. For each sequence, four vegetation types, i.e., grassland, shrubland, secondary forest and primary forest were selected and represented different stages of succession following agricultural abandonment. These vegetation types were regenerated naturally from abandoned croplands distributed in the lower slopes or the valley at different periods, i.e., in the 2000 s, 1980 s and 1950 s for the grassland, shrubland and secondary forest, respectively. The primary forest has not been disturbed over the last 150 years. Soil is calcareous lithosols for all the studied sites with soil texture being silty clay loam or silt loam. Land use history was determined by inquiring the native elder people, therefore uncertainty existed for the estimation of years following agricultural abandonment. The grassland, shrubland and secondary forest were estimated to be 10, 29 and 59 years following agricultural abandonment with ± 5 years in uncertainty. The croplands over the slope were typically planted with maize and were cultivated for over 50 years before abandonment. The croplands were fertilized with animal or human excreta in combination with urea or compound fertilizer with an annual fertilizer N input of about 150 kg ha^−1^. Since most of the cropland close to the reserve was abandoned, only three plots of cropland were chosen for both sequences. Six plots of about 20 m × 20 m were established for each successional stage. The plots (n = 3 for each stage of each sequence) for shrubland, secondary forest and primary forest were distributed within the reserve, with the primary forest located in the core zone and the shrubland and secondary forest located in the outer zone of the reserve. The plots for grassland (n = 3 for each stage of each sequence) and cropland (n = 3 in total) were distributed out of, but close to the reserve. All the plots were selected within a distance less than 6 km. The selected sites were distributed over an elevation range from 300 m to 550 m. The slope of the sites ranged between 10 and 15 degrees. Some soil properties are presented in Table 3.

The field sampling was conducted in July 2014. Following published sampling methodology^36^, soil samples were randomly collected with soil corers at 10 points (>1 m from the trunk of a tree) within each plot. At each sampling point, surface litter (Oi) was collected in an area of 10 cm × 10 cm. Considering the shallow and heterogeneous soil depth on the slope, mineral soil samples to a depth of 15 cm, which represented the average soil depth on the slope in the studied area, were collected after removal of organic layer. The ten soil samples in a plot were mixed as a composite sample. Additional soil cores (4.8 cm in diameter) were collected to determine bulk density (BD).

### Chemical analysis

In the laboratory, roots and stones were picked out using forceps and soils were air dried and passed through a 2-mm mesh sieve. Soil organic carbon (SOC) was measured by wet oxidation with dichromate redox colorimetric method, with which carbonates are not determined^37^. Soil pH (1:2.5 soil/water ratio) was measured with a pH meter (FE20K, Mettler-Toledo, Switzerland). Soil N was analyzed using an elemental analyzer (EA 3000; EuroVector, Italy). Soil total P was determined by acid digestion with a H_2_SO_4_ + HClO_4_ solution^37^. Soil texture was determined using a particle size analyzer (Mastersizer, 2000, Malvern, UK). Exchangeable calcium (Ca), magnesium (Mg), potassium (K) and sodium (Na) were displaced via compulsive exchange in 1 mol L^−1^ ammonium acetate at pH 7.0 and analyzed by inductively coupled plasma atomic emission spectroscopy (ICP-AES)^38^. Final values of the above variables were reported on a dry soil basis, where dry soil denotes soil was dried to constant weight at 105 °C for 24 h.

### Data analysis

SOC content (Mg C ha^−1^) was estimated according to SOC concentration (g C kg^−1^), soil sampling depth (cm), bulk density (g cm^−3^) and fraction of >2 mm fragments^39^,^40^. Two-way ANOVA with LSD test was used to examine the effects of sequence and successional stage and their possible interaction on SOC content. If there is interaction between sequence and successional stage, one way ANOVA was performed to test the difference of SOC content between successional stages. Stepwise multiple linear regression (MLR) was used to identify and evaluate the contributions of predictive variables to SOC dynamics. Classification and regression tree (CART) analysis was adopted to evaluate the influences of independent variables on SOC variance. CART analysis is a non-parametric technique for the sequential partitioning of a dataset composed of a response variable and any number of potential predictor variables^41^. CART is ideally suited for analysis of the relative importance of predictor variables in explaining variation in the response variable and can deal with nonlinear relationships, high-order interactions, and missing values^42^,^43^. Details on CART analysis have been presented elsewhere^41^,^42^,^43^,^44^. Significant difference was presented as *p* < 0.05 unless otherwise pointed out. The statistical analyses were performed using SPSS 16 (SPSS Inc., Chicago, IL, USA).

Dynamics of soil SOC contents with years following agricultural abandonment was fitted with a Chapman function (Eqn 1):


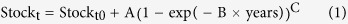


where Content_t_ denotes SOC content after t years following agricultural abandonment; Content_t0_ is SOC content before agricultural abandonment, i.e., SOC content in the cropland; A is the difference between steady state SOC content (Content_st_) and content before agricultural abandonment, therefore SOC content at steady state after agricultural abandonment is the sum of content_t0_ and A; B is the growth constant, and C is the shaping parameter^45^. We assumed that SOC content reached the steady state level if it was 95% of the steady state SOC content. Accordingly, years (T) needed to reach steady state SOC content can be estimated by Eqn 2:


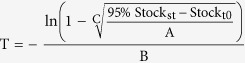


Since the rate of SOC accumulation can’t be directly derived from the above nonlinear fitting, the average rate of SOC accumulation was estimated by dividing A by T.

## Additional Information

**How to cite this article**: Yang, L. *et al*. Soil organic carbon accumulation during post-agricultural succession in a karst area, southwest China. *Sci. Rep.* **6**, 37118; doi: 10.1038/srep37118 (2016).

**Publisher's note:** Springer Nature remains neutral with regard to jurisdictional claims in published maps and institutional affiliations.

## Figures and Tables

**Table 1:** Pearson’s correlation coefficients based on the multiple linear regression analysis.

	Ca	K	Mg	Na	Clay	Silt	Sand	P
K	0.33							
Mg	0.98**	0.29						
Na	−0.02	0.04	−0.07					
Clay	−0.92**	−0.28	−0.91**	0.17				
Silt	0.82**	0.28	0.77**	−0.06	−0.90**			
Sand	0.75**	0.18	0.80**	−0.27	−0.76**	0.45*		
P	0.75**	0.40*	0.72**	0.19	−0.70**	0.65**	0.52**	
SOC	0.94**	0.44*	0.94**	−0.16	−0.93**	0.82**	0.76**	0.76**

*And ** denote p < 0.05 and p < 0.01, respectively.

**Table 2:** Results of stepwise multiple linear regression analyses showing the dependence of SOC on soil physicochemical variables.

Explanatory variable	Coefficient	Partial R^2^	Model R^2^	P value
Ca	0.98	0.891	0.89	0.000
Clay	−1.34	0.025	0.92	0.014
K	28.30	0.021	0.94	0.012

Positive and negative values of coefficients denote positive and negative relationship, respectively, between the explanatory variables and SOC.

**Table 3:** Variation of bulk density (BD, g cm^−3^), pH, soil organic carbon (SOC, g C kg^−1^), total N (g N kg^−1^), total P (g P kg^−1^), exchangeable cations (cmol kg^−1^) and soil texture (%) along with vegetation succession (with a soil depth of 0–15 cm).

		BD	pH	SOC	N	C:N	P	Ca	K	Mg	Na	Clay	Silt	Sand
CR	Mean	1.32^b^	6.31^b^	20.86^b^	1.69^c^	14.16^a^	0.52^c^	8.61^bc^	0.44^ab^	1.95^b^	0.37a	32.66^a^	50.58^b^	16.77^b^
(n = 3)	SE	0.01	0.09	4.45	0.28	0.66	0.03	2.36	0.10	1.27	0.06	1.36	0.65	0.70
GR	Mean	1.37^a^	6.28^b^	19.91^b^	1.85^c^	12.63^a^	0.83^b^	8.21^c^	0.20^c^	2.00^b^	0.40a	36.28^a^	47.23^c^	16.49^b^
(n = 6)	SE	0.01	0.10	1.21	0.11	0.63	0.09	0.61	0.02	0.17	0.03	1.43	0.62	0.95
SH	Mean	1.16^c^	6.88^ab^	42.97^a^	4.40^b^	11.97^a^	1.57^a^	25.58^ab^	0.31^b^	9.42^a^	0.52a	23.48^b^	58.54^a^	17.99^ab^
(n = 6)	SE	0.05	0.31	6.41	0.83	0.77	0.20	6.17	0.02	2.92	0.07	3.03	2.25	0.82
SF	Mean	1.01^c^	7.31^a^	64.59^a^	6.65^a^	13.52^a^	1.37^ab^	33.00^a^	0.30^b^	14.79^a^	0.16b	17.81^b^	59.56^a^	22.63^a^
(n = 6)	SE	0.05	0.15	7.72	0.90	3.46	0.34	3.96	0.03	2.42	0.02	2.36	1.11	2.29
PF	Mean	1.05^c^	6.91^a^	57.88^a^	5.57^ab^	12.07^a^	1.73^a^	28.45^a^	0.53^a^	11.65^a^	0.46a	22.61^b^	57.65^a^	19.74^ab^
(n = 6)	SE	0.03	0.18	5.42	0.42	0.26	0.07	3.27	0.07	1.67	0.04	1.35	0.88	1.51

Note: CR, cropland; GR, grassland; SH, shrubland; SF, secondary forest; PF, primary forest; SE, standard error. Different letters denote significant difference among succession stages at *p* < 0.05 level.
